# Chronoamperometric Ammonium Ion Detection in Water via Conductive Polymers and Gold Nanoparticles

**DOI:** 10.3390/molecules29133028

**Published:** 2024-06-26

**Authors:** Roberta Farina, Silvia Scalese, Domenico Corso, Giuseppe Emanuele Capuano, Giuseppe Andrea Screpis, Maria Anna Coniglio, Guglielmo Guido Condorelli, Sebania Libertino

**Affiliations:** 1 Consiglio Nazionale delle Ricerche-Istituto per la Microelettronica e Microsistemi (CNR-IMM), Strada VIII Z.I., 5, 95121 Catania, Italy; roberta.farina@imm.cnr.it (R.F.); silvia.scalese@imm.cnr.it (S.S.); giuseppeemanuele.capuano@imm.cnr.it (G.E.C.); ma.coniglio@unict.it (M.A.C.); 2 Dipartimento di Scienze Chimiche, Università Degli Studi di Catania, Viale A. Doria 6, 95125 Catania, Italy; guido.condorelli@unict.it; 3 Dipartimento di Scienze Mediche, Chirurgiche e Tecnologie Avanzate “G.F. Ingrassia”, Università Degli Studi di Catania, Via S. Sofia 87, 95123 Catania, Italy

**Keywords:** ammonium ions, polyaniline, screen-printed electrochemical sensor

## Abstract

Monitoring of ammonium ion levels in water is essential due to its significant impact on environmental and human health. This work aims to fabricate and characterize sensitive, real-time, low-cost, and portable amperometric sensors for low NH_4_^+^ concentrations in water. Two strategies were conducted by cyclic voltammetry (CV): electrodeposition of Au nanoparticles on a commercial polyaniline/C electrode (Au/PANI/C), and CV of electropolymerized polyaniline on a commercial carbon electrode (Au/PANIep/C). Au NPs increase the electrical conductivity of PANI and its ability to transfer charges during electrochemical reactions. The electrode performances were tested in a concentration range from 0.35 µM to 7 µM in NH_4_^+^ solution. The results show that the Au/PANI/C electrode performs well for high NH_4_^+^ concentrations (0.34 µM LoD) and worsens for low NH_4_^+^ concentrations (0.01 µM LoD). A reverse performance occurs for the electrode Au/PANIep/C, with a 0.03 µM LoD at low NH_4_^+^ concentration and 0.07 µM LoD at high NH_4_^+^ concentration. The electrodes exhibit a good reproducibility, with a maximum RSD of 3.68% for Au/PANI/C and 5.94% for Au/PANIep/C. In addition, the results of the repeatability tests show that the electrochemical reaction of sensing is fully reversible, leaving the electrode ready for a new detection event.

## 1. Introduction

Ammonia nitrogen (NH_3_ or NH_4_^+^) is a common pollutant in water bodies, originating from agricultural runoff, industrial discharges, and wastewater treatment plants [1]. Elevated levels of ammonium can lead to eutrophication, harmful algal blooms, and adverse effects on aquatic ecosystems and humans [2]. Therefore, monitoring ammonium ion levels in water is important to mitigate the impact on the environment and human health. Various detection tools have been developed for the analysis of ammonia nitrogen in water, including FTIR spectroscopy [3], spectrophotometric methods [1,4], colorimetric pH detection [5,6], ion-selective electrodes [7,8], and other optical methods [9,10,11,12]. The most common sensors are based on a sensitive ion-selective membrane, modified with functional groups, that responds to the presence of NH_4_^+^ in water [8,13,14,15,16]. The disadvantages of these sensors include the need for periodic calibration, the instability of the sensitive membrane over time, and the sensitivity to interferents in the water, which may affect the measurement accuracy. Several methods for monitoring ammonium ions in water have been developed using biological sensors. Microorganisms, such as bacteria and algae, act as bio-indicators to monitor water quality [17,18,19]. Unfortunately, environmental variables can affect biosensors’ performance, and optimal culture conditions are required to ensure correct NH_4_^+^ detection. In addition, although these techniques are specific and sensitive, their use presents several drawbacks, such as the need for sampling and the use of sophisticated, expensive, and time-consuming tools. For these reasons, the development of low-cost, portable, and real-time sensing systems [20,21,22] is a research priority, and electrochemical sensors are the best candidate. Using screen-printing technology, electrodes with reproducible chemical performances can be developed [23,24]. A screen-printed electrochemical cell is composed of three electrodes: a working electrode (WE), which may be functionalized with materials selective towards the analyte; a reference electrode (RE) to ensure the precise application of the WE potential; and a counter electrode (CE) to complete the circuit. This system undergoes an electrochemical reaction that results in changes in current, potential, charge, conductivity, or impedance, which are measured using different electrochemical techniques [25,26,27]. The inks used for printing determine the properties of the electrochemical cell, and the appropriate modification of the working electrode surface plays a key role in the development of sensitive and selective electrochemical sensors for molecule/target substance detection. The working electrode can be modified with different materials, such as metal nanoparticles, metal oxides, organic molecules, and conducting polymers. The sensing mechanism involves changes in electrical conductivity or other properties when NH₃ molecules interact with the surface of the functionalized electrode. Polyaniline (PANI) is considered one of the most versatile conducting polymers, with a wide variety of controllable properties. PANI consists of monomer units built from reduced (y) and oxidized (1 − y) blocks.

**Figure 1:**
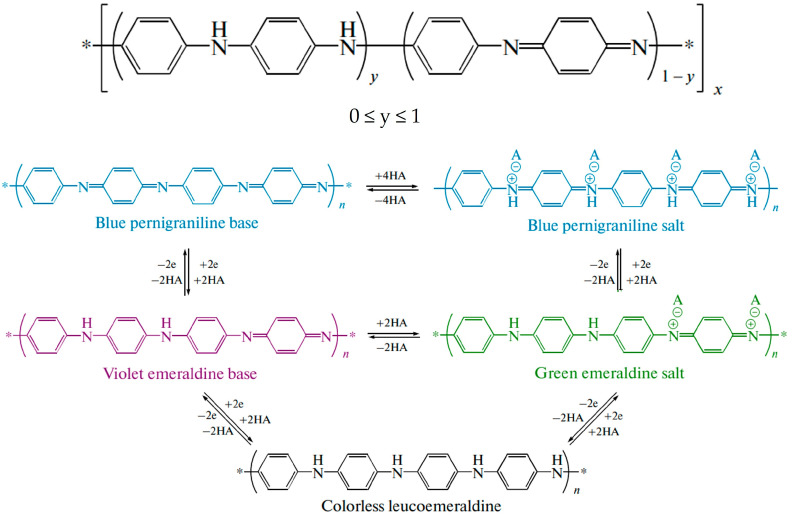
Polyaniline redox state.

The polymer redox state is determined by the value of y (0 ≤ y ≤ 1), which varies from zero to one (Figure 1). At y = 0.5, polyaniline is in the form of emeraldine; y = 0 corresponds to pernigraniline, the fully oxidized form, while y = 1 corresponds to the fully reduced form, leucoemeraldine [28,29]. Pernigraniline and emeraldine may occur as either salts or bases.

The doping process can modify the properties of PANI. Dopants for conductor or semiconductor PANI include inorganic and organic acids such as hydrochloric acid, sulfuric acid, phosphoric acid, formic acid, oleic acid, camphoric sulphonic acid, etc. PANI doping generates high conductivity, mainly due to the increase in carrier concentrations, and leads to the formation of conjugative defects such as solitons, polarons, and bipolarons in the polymer chain that create an electron vacancy mechanism [30]. Proton-doped polyaniline, produced using acids, forms emeraldine salt, which is a highly conducting polymer [31]. Generally, ammonia monitoring is performed for the gas form but can be determined in the ionic form in liquid matrices [7,32,33,34]. Korent et al. developed a sensor for ammonia detection in biological fluids using polyaniline as the conducting polymer and commercial drop-cast gold nanoparticles. They operated at neutral pH using a PBS buffer solution and obtained an LoD of 1.44 μM for an ammonia concentration ranging from 51 μM to 510 mM [30]. Functionalizing PANI with Au NPs promoted the system’s conductivity, which resulted in a better stabilization of the current and sensory performances in the low NH_3_ concentration range. Electrodeposited and drop-cast Au NPs exhibit significant differences in their physical and chemical properties. Electrodeposited gold nanoparticles tend to have a more uniform size and shape than those deposited by drop casting. In addition, electrodeposited Au NPs may also have a higher surface charge density. Therefore, starting from the above-mentioned research, using screen-printed electrodes, the present research aims to develop an ammonium ion detection method in water that is sensitive, selective, inexpensive, and capable of providing a rapid response using a commercial and electropolymerized PANI with Au NPs deposited via cyclic voltammetry (CV) to improve the sensitivity. This study proposes two sensing mechanisms for detecting ammonia as NH_4_^+^. The first uses electropolymerized PANI (PANIep) with commercial screen-printed carbon electrodes (SPCEs), and the second with commercial PANI screen-printed carbon electrodes at acid pH. For both devices, the sensing mechanism is based on the PANI deprotonation reaction, NH_4_^+^ oxidation, and the following PANI reduction and oxidation. The unique properties of polyaniline and gold nanoparticles enable efficient electrochemical signal transduction upon exposure to NH_4_^+^, leading to the accurate quantification of ammonium concentrations in water matrices.

## 2. Results

### 2.1. Morphological Characterization

SEM images were acquired to investigate the morphological difference between the two developed electrodes. 

**Figure 2:**
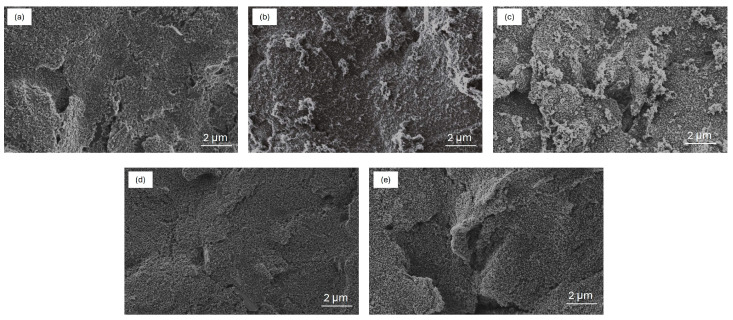
SEM images of (**a**) carbon bare SPE; (**b**) electrodeposited PANI on carbon SPE; (**c**) Au on top of electrodeposited PANI on carbon SPE; (**d**) PANI/C bare SPE; (**e**) Au on top of commercial PANI/C SPE.

Figure 2a shows the surface of the bare C WE characterized by a flake-like structure that is typical of the screen-printing process. After CV electropolymerization, the surface of PANIep/C WE obtained (Figure 2b) shows the nucleation of nanogranular PANIep, growing into nanofibers that do not cover the whole C surface but are located as separate islands. The latter could be a consequence of an uneven C screen-printed electrode surface. The modified Au/PANIep/C WE has a granular morphology composed of particles of different sizes (30–60 nm, see Figure S1b in the Supplementary Materials) and surface textures (Figure 2c). Morphologically, Au NPs do not affect the nanogranular and nanofibrous structure of the PANIep and are uniformly distributed on the surface of the WE obtained, which fully covered the bare surface of the carbon electrode. The commercial bare PANI/C screen-printed WE shows a uniform, interconnected, and porous film of polyaniline on top of the carbon surface (Figure 2d). The conductive PANI film provides increased surface area for the effective electrodeposition of Au NPs. As seen in the SEM images in Figure 2e, a dense and uniform coverage of Au NPs (size 20–30 nm, see Figure S1a in the Supplementary Materials) on the PANI film was obtained. Furthermore, the size of the Au nanoparticles deposited on the commercial PANI/C electrode is smaller than that of the gold nanoparticles deposited on the synthesized PANIep/C electrode. Nanoparticles of different sizes have different surface-to-volume ratios, which may influence their chemical reactivity. In general, smaller Au NPs tend to have a greater surface area to unit mass than larger ones, leading to higher catalytic activity. The size depends on the material of the substrate on which they are electrodeposited. In particular, the micro granular PANIep provides specific anchoring sites for the Au NPs, influencing their distribution and orientation on the carbon surface, while the uniform PANI film on the carbon surface provides a more homogeneous and continuous matrix to support Au NPs. Thus, the exposed carbon surface generates the formation of gold nanoparticles of different sizes. EDX analysis (see Supplementary Materials Figure S2 and Table S1) confirms the Au nanoparticles’ formation on top of both electrodes. The Au distribution on the sample indicates its presence on the whole PANI surface (with a percentage of about 5%) with peaks of 19% where Au nanoparticles are visible in the SEM images.

### 2.2. Electrochemical Characterization of Electropolymerized Polyaniline 

**Figure 3:**
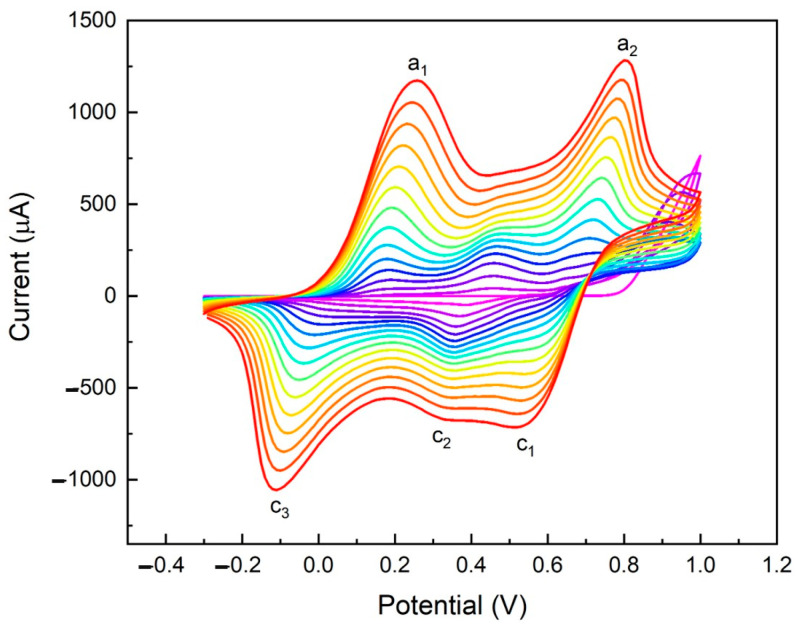
Cyclic voltammograms of polyaniline electropolymerization in 1 M HCl between −0.3 and 1.0 V at 50 mV s^−1^.

The voltammograms in Figure 3 show the typical PANI oxidation (a_1_ = 0.24 V and a_2_ = 0.81 V vs. Ag) and reduction (c_1_ = 0.52 V, c_2_ = 0.30 V, and c_3_ = −0.1 V vs. Ag) peaks [35,36,37]. In the first few cycles of the electropolymerization, the voltammogram shows a PANI oxidation peak at a potential of 0.97 V vs. Ag which is masked by the emeraldine oxidation peak (a_2_ = 0.81 V vs. Ag) [30] after more deposition cycles. In detail, peak a_1_ represents the oxidation of leucoemeraldine to emeraldine, and a_2_ the oxidation of emeraldine to pernigraniline. The peaks c_1_ and c_2_ are relative to the reduction of pernigraniline to emeraldine, and the peak c_3_ is the reduction of emeraldine to leucoemeraldine [28,30,36,37]. 

In an acidic medium, polyaniline exists in a fully reduced form (leucoemeraldine) and undergoes two distinct redox transitions to the half-oxidized form (emeraldine) and the fully oxidized form (pernigraniline). The two oxidation (a_1_, a_2_) and two reduction peaks (c_1_, c_2_) together form two redox couples (a_1_/c_2_ and a_2_/c_1_); specifically, the first redox couple (a_1_/c_2_) is the transition between leucoemeraldine and emeraldine, and the second one (a_2_/c_1_) is the transition between emeraldine and pernigraniline. The final product of the electrochemical polymerization via the CV in the HCl electrolyte is HCl-doped PANI in the form of green emeraldine salt [28,30,36]. The concentration of HCl plays a crucial role in determining the morphology of PANIep. This process of proton doping with HCl helps to delocalize the diiminoquinone-diaminobenzene states trapped in the emerald salt form of polyaniline. This form, called emeraldine base, is neutral but becomes highly electrically conductive after doping with the acid [30]. Therefore, doping with HCl results in the emeraldine salt state of PANI. PANI is a p-type semiconductor indicating most charge carriers as holes. The (ππ) and (ππ*) orbitals serve the role of valence and conduction bands, respectively. The energy difference between (ππ) and (ππ*) is termed as the band gap for the polymer [38,39,40]. Au NPs were electrodeposited via CV on PANI and PANIep. The use of Au NPs during electrode modification offers numerous advantages, such as better diffusion of electroactive compounds, improved catalytic activity, high selectivity, and a higher signal-to-noise (S/N) ratio [38]. In the fabrication of electrochemical sensors, Au NPs can be used to play their role in catalyzing electrochemical reactions and promoting electron transfer [25,41,42,43]. 

The interaction between polyaniline and Au NPs in the sensor design plays a crucial role in promoting the detection of ammonium ions. The synergistic effects of these materials result in improved sensitivity, selectivity, and response time of the sensor toward detecting ammonium ions in water samples [32,44]. 

### 2.3. Chronoamperometric Ammonium Ion Detection in Water

**Figure 4:**
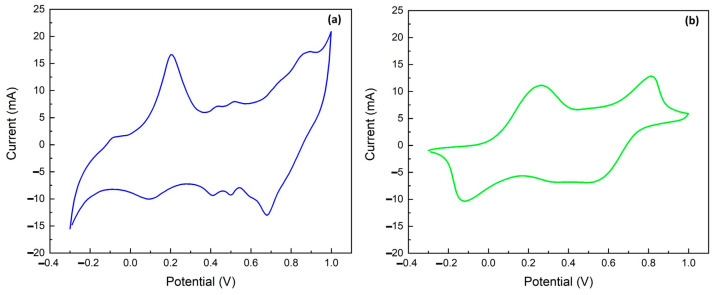
Cyclic voltammograms in 1 M HCl between −0.3 and 1.0 V at 50 mV s^−1^: (**a**) Au/PANI/C; (**b**) Au/PANIep/C.

PANI undergoes redox and protonation reactions during a voltage scan; hence, chronoamperometry (CA) was used as a detection method. Amperometry measures the current generated when a constant potential is applied to the working electrode. The applied voltage causes an oxidation (or reduction) reaction on the electrode surface and the corresponding anodic (or cathodic) current is recorded and correlated with the concentration of the target molecule. To obtain the oxidation (or reduction) potential of the analyte under test, cyclic voltammetry must be performed [24,45,46]. Figure 4 shows the voltammograms for the Au/PANI/C (a) and Au/PANIep/C (b) electrodes.

The PANI electrochemical study was focused on the potential region of the first oxidation peak, a_1_ (i.e., the oxidation of leucoemeraldine to emeraldine), because the pernigraniline form is unstable due to the quinoid-imine structure [28,30]. Therefore, the starting PANI form for NH_4_^+^ detection is the emeraldine salt (protonated half-oxidized form), obtained after the CV polymerization process in HCl. The potentials chosen for electrodes were 0.25 V for Au/PANI/C and 0.35 V for Au/PANIep/C, since these are the potential values after the oxidation process observed through cyclic voltammetry. The switching between leucoemeraldine and emeraldine (a_1_/c_2_) is a diffusion-controlled reaction. NH_4_^+^ forms strong hydrogen bonds with hosts and occupies empty PANI protonation sites [47,48]. The formation of the PANI·NH_4_^+^ complex results in an instantaneous increase in the oxidation current. The application of the potential leads to the oxidation of the ammonium ion, which is the source of electrons and protons for the simultaneous reduction of the emerald base of PANI, resulting in a protonated reduced PANI that simultaneously undergoes an oxidation reaction in the presence of an oxidative potential [30]. After wearing the reductant and the deprotonation of the PANI, the current returns to its initial value and stabilizes. This mechanism produces a transient current response to the presence of NH_4_^+^ in amperometric-mode measurements. Gold nanoparticles on the electrodes improve PANIs’ electrochemical properties by increasing the electrical conductivity of polyaniline and its ability to transfer charges during electrochemical reactions [49]. The electrical quality of the polymer–metal contact is defined by the metal’s Fermi level position and the position of the lowest unoccupied molecular orbital in a polymer [50,51,52]. PANI can form low-resistance ohmic contacts in combination with a metal with a high work function such as Au (5.2 eV), due to its p-type conduction characteristic [53]. 

**Figure 5:**
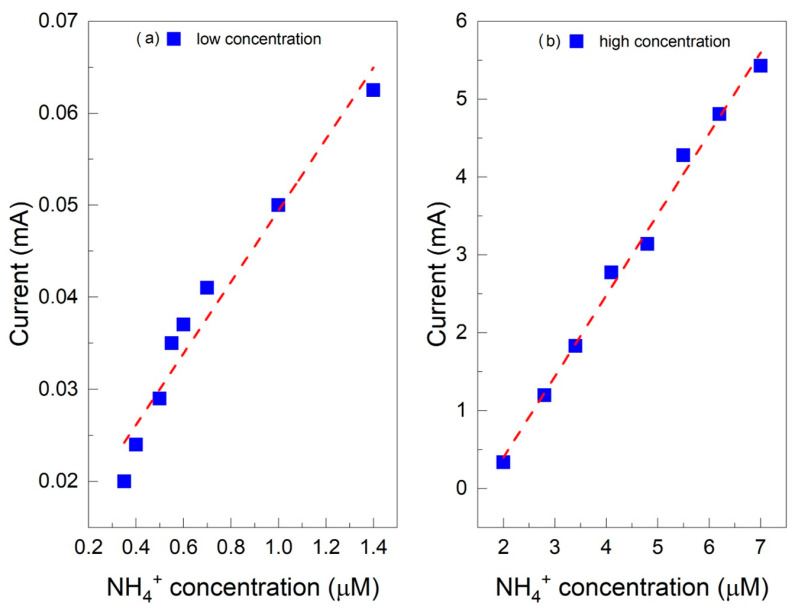
Calibration curves for NH_4_^+^ detection for Au/PANI/C sensor: (**a**) low concentration, from 0.35 µM to 1.5 µM; (**b**) high concentration, from 2 µM to 7 µM.

**Figure 6:**
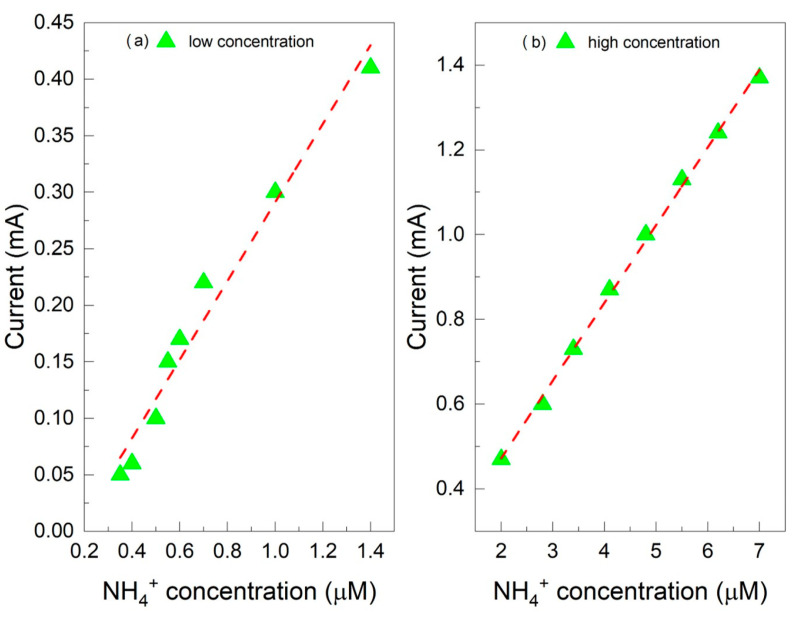
Calibration curves for NH_4_^+^ detection for Au/PANIep/C sensor: (**a**) low concentration, from 0.35 µM to 1.5 µM; (**b**) high concentration, from 2 µM to 7 µM. The red dashed lines correspond to linear fit of the data points.

The effect of different concentrations was studied via amperometry to understand the performance of the two sensors developed for detecting ammonium ions in water. At 25 °C, the pKa of NH_4_^+^ is 9.2, with a pH at which ammonia nitrogen is 50% in NH_3_ form and 50% in NH_4_^+^ form [54]. Therefore, monitoring the ammonium ion is best carried out in neutral and acidic solutions. The relationship between current response and concentration was studied from 0.35 µM to 7 µM NH_4_^+^ in 1 M HCl solution. Figure 5a,b show the amperometric measurement results for the Au/PANI/C electrode. The calibration graphs are linear in two ranges, from 0.35 µM to 1.5 µM with a sensitivity of 0.03 mA/µM (Figure 5a) and from 2 µM to 7 µM with a sensitivity of 1.09 mA/µM (Figure 5b). Again, for the Au/PANIep/C electrode, the calibration graphs are linear in two ranges, from 0.35 µM to 1.5 µM with a sensitivity of 0.34 mA/µM (Figure 6a) and from 2 µM to 7 µM with a sensitivity of 0.18 mA/µM (Figure 6b). The calibration curves for the PANI-NH_3_ detection system were split as previously achieved in the literature [30,55,56].

**Figure 7:**
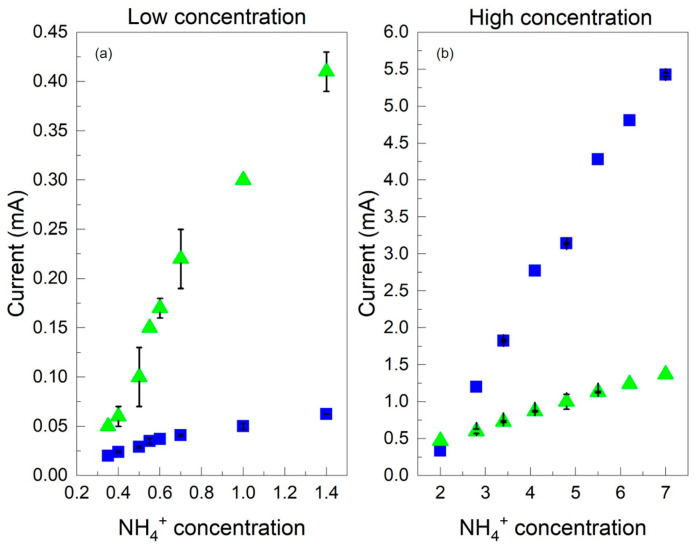
Calibration curves for NH_4_^+^ detection for Au/PANIep/C (green triangles) and Au/PANI/C (blue squares) electrodes: (**a**) low concentration from 0.35 µM to 1.5 µM; (**b**) high concentration from 2 µM to 7 µM.

The results show that the Au/PANI/C electrode performs better for high NH_4_^+^ concentrations and worse for low NH_4_^+^ concentrations than the Au/PANIep/C WE. The opposite behavior is observed for the latter electrode (Figure 7). This phenomenon can be explained by considering the properties of the two electrodes. Specifically, the commercial fully PANI-coated carbon electrode works poorly with low concentrations of ammonium ions, since the PANI is HCl-undoped; therefore, it may be difficult for NH_4_^+^ to reach the electrode surface and participate in the detection process due to the diffusion. Instead, it works well for high NH_4_^+^ concentrations since the increased presence of ammonium ions may exceed the diffusion limits and favor the interaction between the ions and the polyaniline. On the other hand, the C electrode coated with nanogranular PANIep works well for low concentrations of ammonium ions because the polyaniline allows greater accessibility of NH_4_^+^ to the electrode surface, but the sensor works poorly for high ammonium ion concentrations because the nanogranular PANI can quickly saturate and become unable to handle high amounts of NH_4_^+^. This could be a consequence of the complete occupation of the empty protonation sites of PANI by NH_4_^+^. In summary, the distribution of polyaniline on the carbon electrode can significantly affect the sensitivity and dynamic range of the sensor for amperometric detection of ammonium ions for a wide concentration range. The electrode exhibits good reproducibility with a maximum relative standard deviation (RSD) of 5.94% for Au/PANIep/C and 3.68% for Au/PANI/C.

### 2.4. Repeatability Test

**Figure 8:**
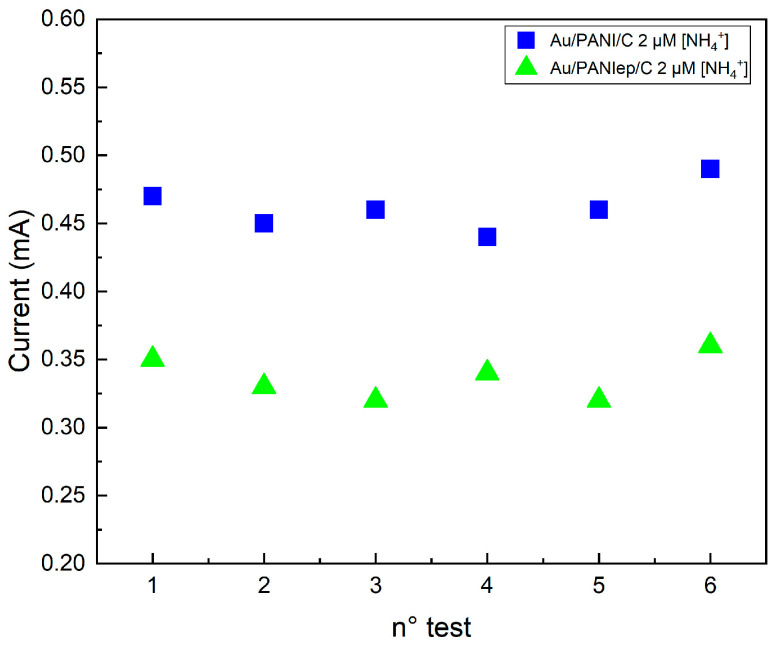
Repeatability test for Au/PANIep/C electrode (green squares), and Au/PANI/C electrode (blue dots) at 2 µM NH_4_^+^ concentration. The oxidation peak current of the NH_4_^+^ was acquired from the same sample by repeating the measurement 6 times.

The repeatability test was carried out using the same sensors, Au/PANI/C and Au/PANIep/C, for six consecutive measurements using the same NH_4_^+^ solution. A concentration of 2 mM was chosen, above the regime change threshold in the sensitivity of both electrodes. Each electrode was washed with distilled water and wiped with a paper towel between two measurements. The standard deviation (SD) was calculated by comparing the subsequent measurements’ current peak with the first one. Figure 8 shows the amperometric response of sequential injections of 2 µM NH_4_^+^ for the Au/PANI/C electrode (blue dots) and the Au/PANIep/C electrode (green squares). After each current stabilization, the sequential injection gave similar maximum oxidation currents (0.47 mA ± 0.02 mA for Au/PANIep/C and 0.35 mA ± 0.02 mA for Au/PANI/C). 

The calculated SD was 0 µA, 14.14 µA, 21 µA, 7.10 µA, 21.20 µA, and 7 µA for Au/PANIep/C, and 0 µA, 14 µA, 6.9 µA, 20.1 µA, 7 µA, and 14.1 µA for Au/PANI/C (Table 1). These results show that the electrochemical detection mechanism is fully reversible, confirming the complete recovery of the PANIep after each measurement and a compact electrode after cleaning and wiping.

## 3. Materials and Methods

Ammonium chloride (NH_4_Cl), phosphate-buffered saline (PBS), hydrochloric acid (HCl), aniline (Ph-NH_2_), and chloroauric acid (HauCl_4_) were purchased from Merck KGaA (Headquarters in Darmstadt, Germany) and used without further purification. Milliq water (resistivity of ~18.2 Ω cm) by Simplicity UV (Millipore, by Merk, Headquarters in Darmstadt, Germany) was used in all solutions. SPEs (screen-printed carbon electrodes, cod. Ref. 150, and screen-printed polyaniline electrodes, cod. Ref 110PANI) were bought from Metrohm DropSens s.r.l. (Origgio, VA, Italy). Electrodeposition of Au and PANI, as well as all electrochemical measurements, were performed using the Palmsens4 electrochemical workstation by PalmSens BV (C-PS4-BP.F2.10, GA Houten, The Netherlands). Scanning electron microscopy (SEM) images were obtained using a ZEISS FE-SEM SUPRA 35 (Carl Zeiss AG, Jena, Germany). 

### Electrode Fabrication

Two amperometric sensors were fabricated for the detection of ammonium ions in water by using two commercial SPEs. Specifically, one SPE has a C screen-printed working electrode, while the second has a PANI/C screen-printed working electrode. PANI electrodeposition on the C working electrode (4 mm diameter) was performed using 0.1 M aniline monomer in a 1 M HCl solution. Classically, hydrochloric acid (HCl) with sulfuric acid (H_2_SO_4_) and chloric acid (HClO_3_) are used as dopants in conductive PANI synthesis. In acid doping, the basic action of polymeraldine with HCl induces protons at immune spots and gives polymeraldine salt. Low temperatures and high dopant (HCl) concentrations favor a faster polymerization process and the production of a thicker polymer film; therefore, the electropolymerization of aniline was carried out at room temperature with a low concentration of HCl (1 M). Electrodeposition was performed on the WE by CV using 15 cycles of scan in a potential range from −0.3 V to 1.0 V, with a scan rate of 0.05 Vs^−1^. 

The commercial polyaniline-modified screen-printed carbon electrode (PANI/C) cell consisted of three electrodes: the working electrode was made of carbon ink modified with polyaniline, with a counter electrode of carbon ink. In contrast, silver ink was used as the reference electrode. 

Au electrodeposition on the fabricated PANIep/C working electrode and commercial PANI/C electrodes (4 mm diameter) was performed using 0.1 M HAuCl_4_ in a 1 M HCl electrolyte solution. Electrodeposition was performed by CV using 5 cycles of scan in a potential range from 0.0 V to 1.0 V, with a scan rate of 0.05 Vs^−1^. 

**Figure 9:**
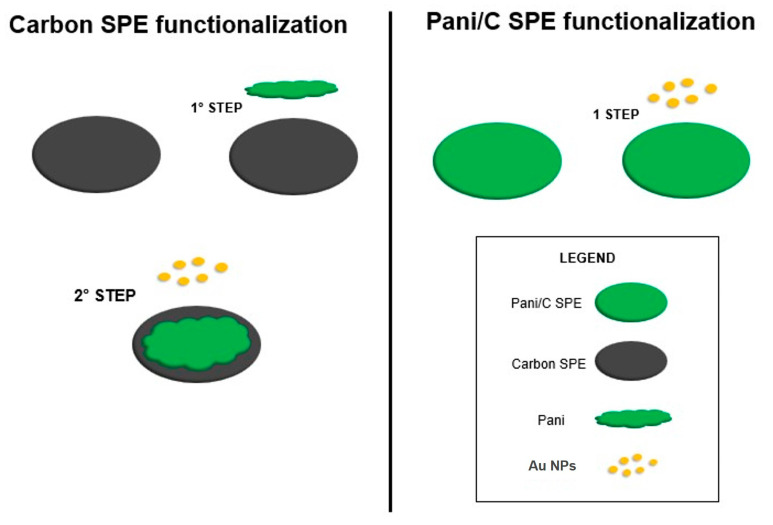
Functionalization scheme process of two different electrodes.

Figure 9 summarizes the functionalization process of two different electrodes: Au on top of the electrodeposited PANIep on the carbon SPE (Au/PANIep/C) and the electrode with Au on top of the PANI/C commercial SPE (Au/PANI/C).

## 4. Conclusions

In this work, two amperometric sensors for NH_4_^+^ concentration in water were developed. Two strategies were conducted for their fabrication by cyclic voltammetry electrodeposition of Au NPs on (i) a commercial PANI/C electrode (Au/PANI/C) and (ii) after CV electropolymerization of a PANIep/C electrode (Au/PANIep/C). In the last sensor, PANI CV electropolymerization produces the growth of nanogranular PANIep into separate nanofibers on the C surface. Indeed, the commercial bare PANI/C electrode shows a uniform, interconnected, and porous film of polyaniline on the carbon surface. The size of the Au NPs deposited on the commercial PANI/C electrode is smaller than that of the Au NPs deposited on the synthesized PANIep/C electrode. Smaller Au NPs have a wider surface area to unit mass than larger ones, leading to higher catalytic activity. For both devices, Au NPs increase the electrical conductivity of polyaniline and its ability to transfer charges during electrochemical reactions. The electrodes’ performances were tested in a concentration range from 0.35 µM to 7 µM NH_4_^+^ in 1 M HCl solution; the results show that the Au/PANI/C electrode performs better for high NH_4_^+^ concentrations (0.34 µM LoD) and worse for low NH_4_^+^ concentrations (0.01 µM LoD). Opposite behavior occurs for the Au/PANIep/C electrode, with a 0.03 µM LoD for low NH_4_^+^ concentration and 0.07 µM LoD for high NH_4_^+^ concentration. The distribution of polyaniline on the carbon electrode can significantly affect the sensitivity and dynamic range of the sensor for amperometric detection of NH_4_^+^. In conclusion, the electrodes exhibit good reproducibility (with a maximum RSD of 5.94% for Au/PANIep/C and 3.68% for Au/PANI/C), and the results of the repeatability tests show full WE recovery after each measurement. The proposed sensors can be easily implemented in precision agriculture as a low-cost, fast, specific, and sensitive method for ammonium ion detection in irrigation water.

## Figures and Tables

**Table 1:** Calculated SD for repeatability tests.

Electrodes	SD
Au/PANIep/C	0 µA
	14.14 µA
	21 µA
	7.10 µA
	21.20 µA
	7 µA
Au/PANI/C	0 µA
	14 µA
	6.9 µA
	20.1 µA
	7 µA
	14.1 µA

## Data Availability

The raw data supporting the conclusions of this article are included in the paper and Supplementary Materials figures and tables.

## Supplementary Materials

The following supporting information can be downloaded at: https://www.mdpi.com/article/10.3390/molecules29133028/s1, Figure S1: SEM images of Au on top of (**a**) commercial PANI/C SPE; (**b**) electrodeposited PANI on Carbon SPE; Figure S2: EDX analysis on top of an Au nanoparticle (grey spectrum) on a surface region where Au nanoparticles were not visible by SEM (red spectrum); Table S1: Element’s relative abundance on the WE Au/PANIep/C.
