# Supplementary material for: Chronoamperometric Ammonium Ion Detection in Water via Conductive Polymers and Gold Nanoparticles
Source: Molecules. 2024 Jun 26;29(13):3028. doi: 10.3390/molecules29133028 (PMC11243345; doi:10.3390/molecules29133028)
Supplement: Supplementary file 1 [file molecules-29-03028-s001.zip › molecules-3070715-supplementary.pdf]

Supplementary Material

Chronoamperometric Ammonium Ion Detection in Water via Conductive Polymers and Gold Nanoparticles

Roberta Farina [1,2], Silvia Scalese [1], Domenico Corso [1,*], Giuseppe Emanuele Capuano [1], Giuseppe Andrea Screpis [3], Maria Anna Coniglio [1,3], Guglielmo Guido Condorelli [2] and Sebania Libertino [1,*]

[1] Consiglio Nazionale Delle Ricerche–Istituto per la Microelettronica e Microsistemi (CNR-IMM), Strada VIII Z.I., 5, 95121 Catania, Italy; roberta.farina@imm.cnr.it (R.F.); silvia.scalese@imm.cnr.it (S.S.); giuseppeemanuele.capuano@imm.cnr.it (G.E.C.); ma.coniglio@unict.it (M.A.C.)
[2] Dipartimento di Scienze Chimiche, Università Degli Studi di Catania, Viale A. Doria 6, 95125 Catania, Italy; guido.condorelli@unict.it
[3] Dipartimento di Scienze Mediche, Chirurgiche e Tecnologie Avanzate "G.F. Ingrassia", Università Degli Studi di Catania, via S. Sofia 87, 95123 Catania, Italy
* Correspondence: domenico.corso@imm.cnr.it (D.C.); sebania.libertino@imm.cnr.it (S.L.)

3.1. Morphological characterization

SEM images of Au deposited on top of electrodeposited PANI (Figure S1a) and commercial PANI SPE are hereafter shown. Images indicate the good PANI coverage with Au nanoparticles and the nanoparticle sizes.

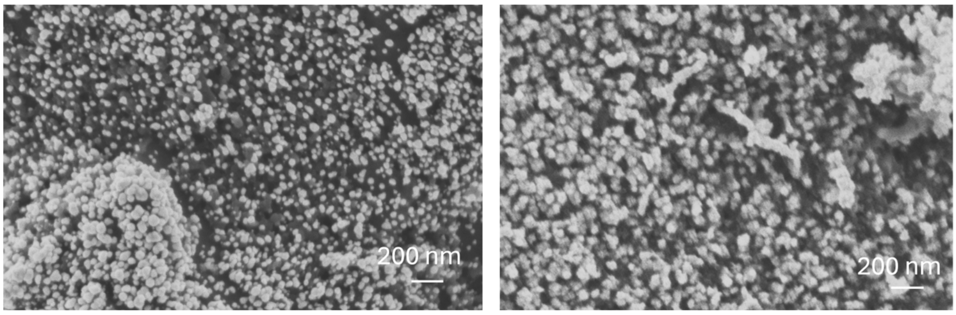

(a) (b)

Figure S1. SEM images of Au on top of **(a)** commercial PANI/C SPE; **(b)** electrodeposited PANI on Carbon SPE.

Gold nanoparticles have different sizes on the two electrodes. The conductive PANI film commercially produced has a uniform coverage of Au NPs having sizes of 20-30 nm, see Figure S1a. In contrast, on the modified PANIep/C WE the Au nanoparticles deposit to a size of 30-60 nm, and larger aggregates are observed on the surface as shown in Figure S1b.

EDX measurements were performed on an Au particle (gray spectrum in Fig. S2) and in a region where Au nanoparticles (Au NP) were not visible (red spectrum in Fig. S2, "clean" surface in Table S1). The graph inspection indicates the presence of C, Au, N, Cl, and O. Each element's relative abundance is reported in Table S1 for the two inspected regions. Au is present also on the "clean" surface but at low relative abundance (5.1%) compared to the Au NP (19.3%) indicating that nanoparticles not visible by SEM, or even single atoms, are dispersed on top of the surface. PANI has one N atom for each benzene ring (six C atoms), and it is HCl-doped. Finally, O presence is due to the C electrode fabrication process. These elements' relative abundances are higher where Au abundance is lower, as expected.

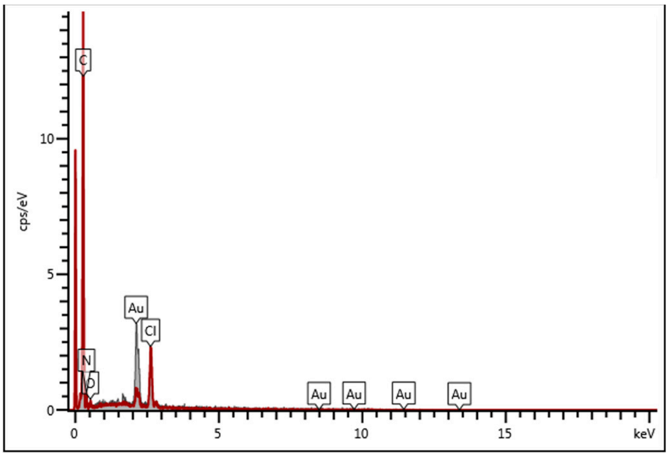

Figure S2. EDX analysis on top of an Au nanoparticle (grey spectrum) on a surface region where Au nanoparticles were not visible by SEM (red spectrum).

Table S1. Element's relative abundance on the WE Au/PANIep/C.

Element	Wt% on top of Au NP	Wt% on a "clean" surface
C	62.4	72.4
Au	19.3	5.1
N	11.8	14.1
Cl	4.9	6.3
O	1.7	2.0
